# Supplementary material for: Network-Based Approaches Reveal Potential Therapeutic Targets for Host-Directed Antileishmanial Therapy Driving Drug Repurposing
Source: Microbiol Spectr. 2021 Oct 20;9(2):e01018-21. doi: 10.1128/Spectrum.01018-21 (PMC8528132; doi:10.1128/Spectrum.01018-21)
Supplement: SUPPLEMENTAL FILE 5 — Supplemental material. Download SPECTRUM01018-21_Supp_5_seq10.pdf, PDF file, 0.1 MB [file spectrum01018-21_supp_5_seq10.pdf]

Supplementary tables

Supplementary table 1. non-coding RNAs differentially expressed in response to *Leishmania major* infection in human macrophages

ncRNA class	4 hpi	24 hpi	48 hpi	72 hpi
lncRNA	407/309	159/322	120/272	34/112
miRNA	8/2	-/1	1/1	NA
miscRNA	6/3	2/-	1/-	1/-
MtrRNA	1/-	1/-	NA	NA
snoRNA	5/-	3/1	-/1	NA
snRNA	1/-	-/1	NA	NA

Numbers corresponds to upregulated/downregulated ncRNAs

Supplementary Table 2. Description of the reference network and context-specific GRNs of *Leishmania major* infected macrophages and non-infected macrophages.

	Total Genes	Edges	Nodes as TFs
Reference GRN (DoRothEA)	20244	486751	1395
4 h post infection	19750	343072	990
24 h post infection	19750	343201	987
48 h post infection	19725	341608	987
72 h post infection	19718	339390	974
4 h non-infected	19760	344812	999
24 h non-infected	19748	342770	993
48 h non-infected	19595	342732	992
72 h non-infected	19610	345596	998

Supplementary Table 3. Nodes and edges only present in *Leishmania major* infected macrophage specific context networks

Comparison	Edge	Total nodes	Nodes as TFs
4 hpi	160	63	11
24 hpi	246	244	21
48 hpi	154	155	20
72 hpi	50	52	9

Supplementary Table 4. Potential therapeutic gene targets and all their direct connection to drugs and reactome pathways

Gene	DrugBank ID	Drug name	Drug group	Pathway in Reactome	Pathway name
**AR**	DB01406	Danazol	approved	R-HSA-418990	Adherens junctions interactions
	DB06710	Methyltestosterone	approved	R-HSA-9607240	FLT3 Signaling
	DB09389	Norgestrel	approved		
	DB13155	Esculin	approved		
	DB13943	Testosterone cypionate	approved		
	DB13944	Testosterone enanthate	approved		
	DB00717	Norethisterone	approved		
	DB00621	Oxandrolone	approved, investigational		
	DB00624	Testosterone	approved, investigational		
	DB13946	Testosterone undecanoate	approved, investigational		
	DB06718	Stanozolol	approved, vet-approved		
	DB00396	Progesterone	approved, vet-approved		
	DB01128	Bicalutamide	approved		
	DB01395	Drospirenone	approved		
	DB00421	Spironolactone	approved		
	DB11219	Enzacamene	approved		
	DB09086	Eugenol	approved		
	DB00648	Mitotane	approved		
	DB09123	Dienogest	approved		
	DB11619	Gestrinone	approved		
	DB00499	Flutamide	approved, investigational		
	DB00665	Nilutamide	approved, investigational		
	DB04839	Cyproterone acetate	approved, investigational		
	DB01428	Oxybenzone	approved, investigational		
	DB11064	Homosalate	approved, investigational		
	DB00255	Diethylstilbestrol	approved, investigational		
	DB11901	Apalutamide	approved, investigational		
	DB12941	Darolutamide	approved, investigational		
	DB12499	Clascoterone	approved, investigational		
	DB00367	Levonorgestrel	approved, investigational		
	DB01026	Ketoconazole	approved, investigational		
	DB08899	Enzalutamide	approved		
	DB06713	Norelgestromin	approved, investigational		
	DB00957	Norgestimate	approved, investigational		
	DB02266	Flufenamic acid	approved		
	DB08867	Ulipristal	approved		
	DB00655	Estrone	approved		
	DB00675	Tamoxifen	approved		
	DB01063	Acetophenazine	approved		
	DB00623	Fluphenazine	approved		
	DB09371	Norethynodrel	approved		
	DB01608	Periciazine	approved, investigational		

	DB08604	Triclosan	approved, investigational		
**CCND1**	DB11718	Encorafenib	approved, investigational	R-HSA-111457	Release of apoptotic factors from the
	DB01169	Arsenic trioxide	approved, investigational	R-HAS-1257604	mitochondria
	DB00945	Acetylsalicylic acid	approved, vet-approved	R-HSA-194840	PI3K/AKT Signaling
				R-HSA-3000178	Rho GTPase cycle
				R-HSA-6804756	ECM proteoglycans
					Regulation of TP53 Activity through
				R-HSA-6807004	Phosphorylation
				R-HSA-8851805	Negative regulation of MET activity
				R-HSA-9617828	MET activated RAS signalling
					FOXO-mediated transcription of cell cycle
					genes
**CD44**	DB08818	Hyaluronic acid	approved, vet-approved	R-HSA-6802946	Signaling by moderate kinase activity BRAF
					mutants
				R-HSA-5693571	Nonhomologous End-Joining (NHEJ)
				R-HSA-5693548	Sensing of DNA Double Strand Break
				R-HSA-5621575	CD209 (DC-SIGN) signalling
				R-HSA-5621480	Dectin-2 family
				R-HSA-2162123	Synthesis of Prostaglandins (PG) and
					Thromboxanes (TX)
**JUN**	DB00570	Vinblastine	approved	R-HSA-2122947	NOTCH1 Intracellular Domain Regulates
	DB01029	Irbesartan	approved, investigational		Transcription
	DB01169	Arsenic trioxide	approved, investigational	R-HSA-3299685	Detoxification of Reactive Oxygen Species
	DB00210	Adapalene	approved	R-HSA-375165	NCAM signalling for neurite out-growth
				R-HSA-446107	Type I hemidesmosome assembly
				R-HSA-5625886	Activated PKN1 stimulates transcription of
					AR (androgen receptor) regulated genes
					KLK2 and KLK
				R-HSA-5628897	TP53 Regulates Metabolic Genes
				R-HSA-8866376	Reelin signalling pathway
				R-HSA-8873719	RAB geranylgeranylation
				R-HSA-8875791	MET activates STAT3
				R-HSA-9627069	Regulation of the apoptosome activity
**MT2A**	DB00515	Cisplatin	approved	R-HSA-389356	CD28 co-stimulation
	DB00958	Carboplatin	approved	R-HSA-5693571	Nonhomologous End-Joining (NHEJ)
	DB00526	Oxaliplatin	approved, investigational		
	DB01593	Zinc	approved, investigational		
	DB14487	Zinc acetate	approved, investigational		
	DB14533	Zinc chloride	approved, investigational		
**PDGFRA**	DB10772	Foreskin keratinocyte		R-HSA-143355	Signaling by SCF-KIT
	DB00619	Imatinib		R-HSA-3371497	HSP90 chaperone cycle for SHRs
	DB06043	Olaratumab			
	DB06595	Midostaurin			

	DB06589	Pazopanib			
	DB08896	Regorafenib			
	DB09079	Nintedanib			
	DB14840	Ripretinib			
	DB01268	Sunitinib			
	DB08901	Ponatinib			
	DB09078	Lenvatinib			
	DB12010	Fostamatinib			
	DB12147	Erdafitinib			
	DB00102	Becaplermin			
**PTGS2**	DB09212	Loxoprofen	approved	R-HSA-6804757	Regulation of TP53 Degradation
	DB11071	Phenyl salicylate	approved	R-HSA-6804756	Regulation of TP53 Activity through Phosphorylation
	DB11323	Glycol salicylate	approved		
	DB11201	Menthyl salicylate	approved		
	DB13783	Acemetacin	approved, investigational		
	DB09216	Tolfenamic acid	approved, investigational		
	DB09214	Dexketoprofen	approved, investigational		
	DB00887	Bumetanide	approved		
	DB00035	Desmopressin	approved		
	DB00041	Aldesleukin	approved		
	DB08819	Tafluprost	approved		
	DB00884	Risedronic acid	approved, investigational		
	DB00360	Sapropterin	approved, investigational		
	DB00784	Mefenamic acid	approved		
	DB00991	Oxaprozin	approved		
	DB00244	Mesalazine	approved		
	DB00316	Acetaminophen	approved		
	DB00461	Nabumetone	approved		
	DB00465	Ketorolac	approved		
	DB00469	Tenoxicam	approved		
	DB00500	Tolmetin	approved		
	DB00573	Fenoprofen	approved		
	DB00963	Bromfenac	approved		
	DB01050	Ibuprofen	approved		
	DB01399	Salsalate	approved		
	DB01600	Tiaprofenic acid	approved		
	DB00795	Sulfasalazine	approved		
	DB01419	Antrafenine	approved		
	DB01401	Choline magnesium	approved		
	DB00233	trisalicylate	approved		
	DB06774	Aminosalicylic acid	approved		
	DB00515	Capsaicin	approved		
	DB08910	Cisplatin	approved		
	DB08439	Pomalidomide	approved		
	DB11079	Parecoxib	approved		
	DB00482	Trolamine salicylate	approved, investigational		
	DB00605	Celecoxib	approved, investigational		
	DB00712	Sulindac	approved, investigational		
	DB00328	Flurbiprofen	approved, investigational		

	DB00861	Indomethacin	approved, investigational		
	DB01014	Diflunisal	approved, investigational		
	DB01283	Balsalazide	approved, investigational		
	DB01628	Lumiracoxib	approved, investigational		
	DB00554	Etoricoxib	approved, investigational		
	DB06725	Piroxicam	approved, investigational		
	DB06802	Lornoxicam	approved, investigational		
	DB01435	Nepafenac	approved, investigational		
	DB09213	Antipyrine	approved, investigational		
	DB06736	Dexibuprofen	approved, investigational		
	DB09061	Aceclofenac	approved, investigational		
	DB00749	Cannabidiol	approved, investigational, vet-		
	DB00936	Etodolac	approved		
	DB00720	Salicylic acid	approved, investigational, vet-		
	DB00812	Clodronic acid	approved		
	DB00586	Phenylbutazone	approved, investigational, vet-		
	DB00788	Diclofenac	approved		
	DB00814	Naproxen	approved, vet-approved		
	DB00939	Meloxicam	approved, vet-approved		
	DB00945	Meclofenamic acid	approved, vet-approved		
	DB01009	Acetylsalicylic acid	approved, vet-approved		
	DB00620	Ketoprofen	approved, vet-approved		
	DB01395	Triamcinolone	approved, vet-approved		
	DB00480	Drospirenone	approved, vet-approved		
	DB00773	Lenalidomide	approved, vet-approved		
	DB00250	Etoposide	approved		
	DB02266	Dapsone	approved		
	DB11327	Flufenamic acid	approved		
		Dipyrithione	approved, investigational		
			approved		
**VEGFA**	DB10772	Foreskin keratinocyte	approved	R-HSA-201451	Signaling by BMP
	DB04895	Pegaptanib	approved, investigational	R-HSA-210991	Basigin interactions
	DB08885	Aflibercept	approved	R-HSA-4090294	SUMOylation of intracellular receptors
	DB05294	Vandetanib	approved	R-HSA-5661231	Metallothioneins bind metals
	DB06779	Dalteparin	approved	R-HSA-6804756	Regulation of TP53 Activity through
	DB01017	Minocycline	approved, investigational		Phosphorylation
	DB14864	Brolucizumab	approved, investigational		
	DB01136	Carvedilol	approved, investigational		
	DB01120	Gliclazide	approved		
	DB01270	Ranibizumab	approved		
	DB03088	Pidolic acid	approved, investigational		
	DB00112	Bevacizumab	approved, investigational		
