# Supplementary material for: Network-Based Approaches Reveal Potential Therapeutic Targets for Host-Directed Antileishmanial Therapy Driving Drug Repurposing
Source: Microbiol Spectr. 2021 Oct 20;9(2):e01018-21. doi: 10.1128/Spectrum.01018-21 (PMC8528132; doi:10.1128/Spectrum.01018-21)
Supplement: SUPPLEMENTAL FILE 6 — Supplemental material. Download SPECTRUM01018-21_Supp_6_seq11.pdf, PDF file, 7.6 MB [file spectrum01018-21_supp_6_seq11.pdf]

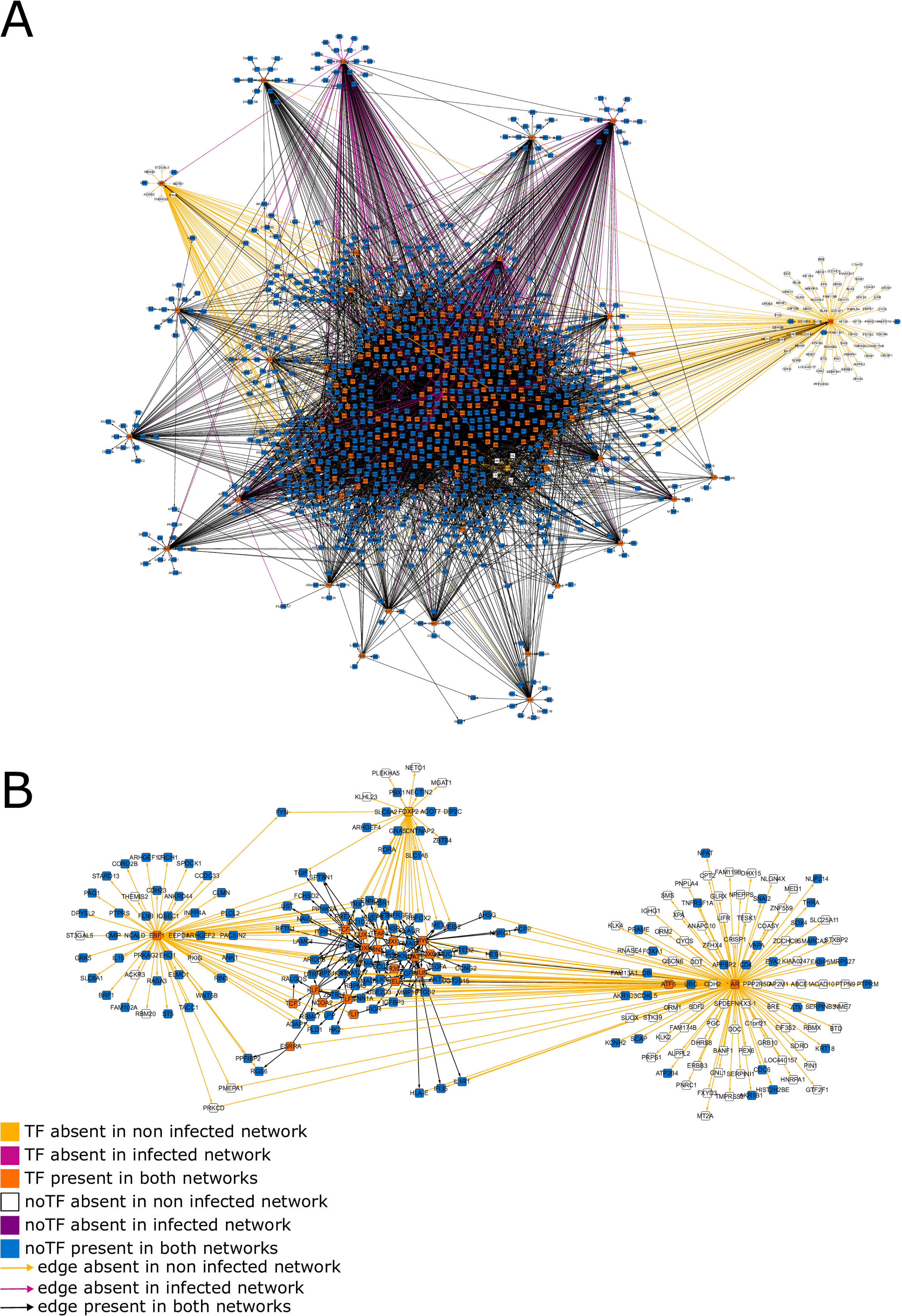

A
B
FOXP2
EBF1
AR
ESRRA
ATF6
TF absent in non infected network
TF absent in infected network
TF present in both networks
noTF absent in non infected network
noTF absent in infected network
noTF present in both networks
edge absent in non infected network
edge absent in infected network
edge present in both networks

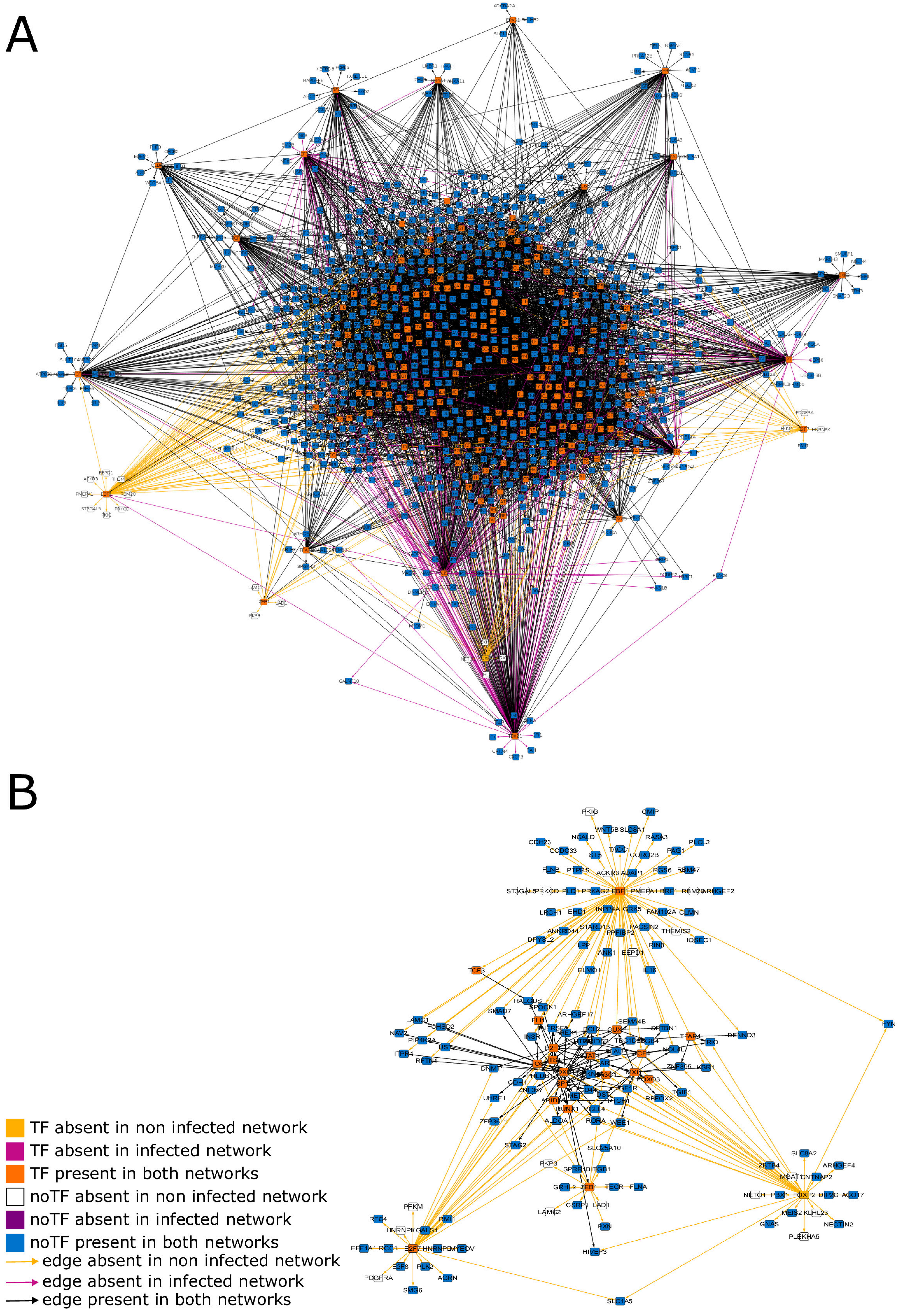
A
B
PKIG
OMIP
WNT5B
SLC6A1
CDH23
NCALD
RASA3
PLCL2
CCDC33
ST5
TACC1
CORO2B
PAG1
FLNB
PTPRS
ACKR3
ADAP1
RGS6
RBM47
ST3GAL5
PRKCD
PLD1
PRKAG2
EBF1
PMEPA1
BRF1
RBM20
ARHGEF2
LRCH1
EHD1
INPP4A
GRK5
FAM102A
CLMN
ANKRD44
STARD13
PACSIN2
THEMIS2
PPFIBP2
DPYSL2
LPP
RIN3
IQSEC1
ANK1
EEPD1
TCF3
ELMO1
IL16
RALGDS
SPOCK1
SMAD7
ARHGEF17
LAMC1
SEMA4B
SPTBN1
FCHSD2
NAV2
DENND3
FYN
PIP4K2A
ITPR1
RFTN1
DNMT1
INSR
CDH1
UHRF1
ZNF367
ZFP36L1
STAG2
ALDOA
RORA
WEE1
RUNX1
VGLL4
PTCH1
RBFOX2
TGIF1
FOXO3
ZNF395
KSR1
SLC25A10
PKP3
SPRR1B
ITGB1
GRHL2
ZEB1
TEC
FLNA
CSRP1
LAD1
LAMC2
PXN
HIVEP3
SLC8A2
ZBTB4
ARHGEF4
MGAT
CNTNAP2
NETO1
PBX1
FOXP2
DIP2C
ACOT7
MEIS2
KLHL23
NECTIN2
GNAS
PLEKHA5
PFKM
RFC4
RMI1
HNRNPK
GALS1
EEF1A1
RCC1
E2F7
HNRNPD
MYEOV
E2F8
PLK2
PDGFRA
AGRN
SMC6
SLC1A5
TF absent in non infected network
TF absent in infected network
TF present in both networks
noTF absent in non infected network
noTF absent in infected network
noTF present in both networks
edge absent in non infected network
edge absent in infected network
edge present in both networks

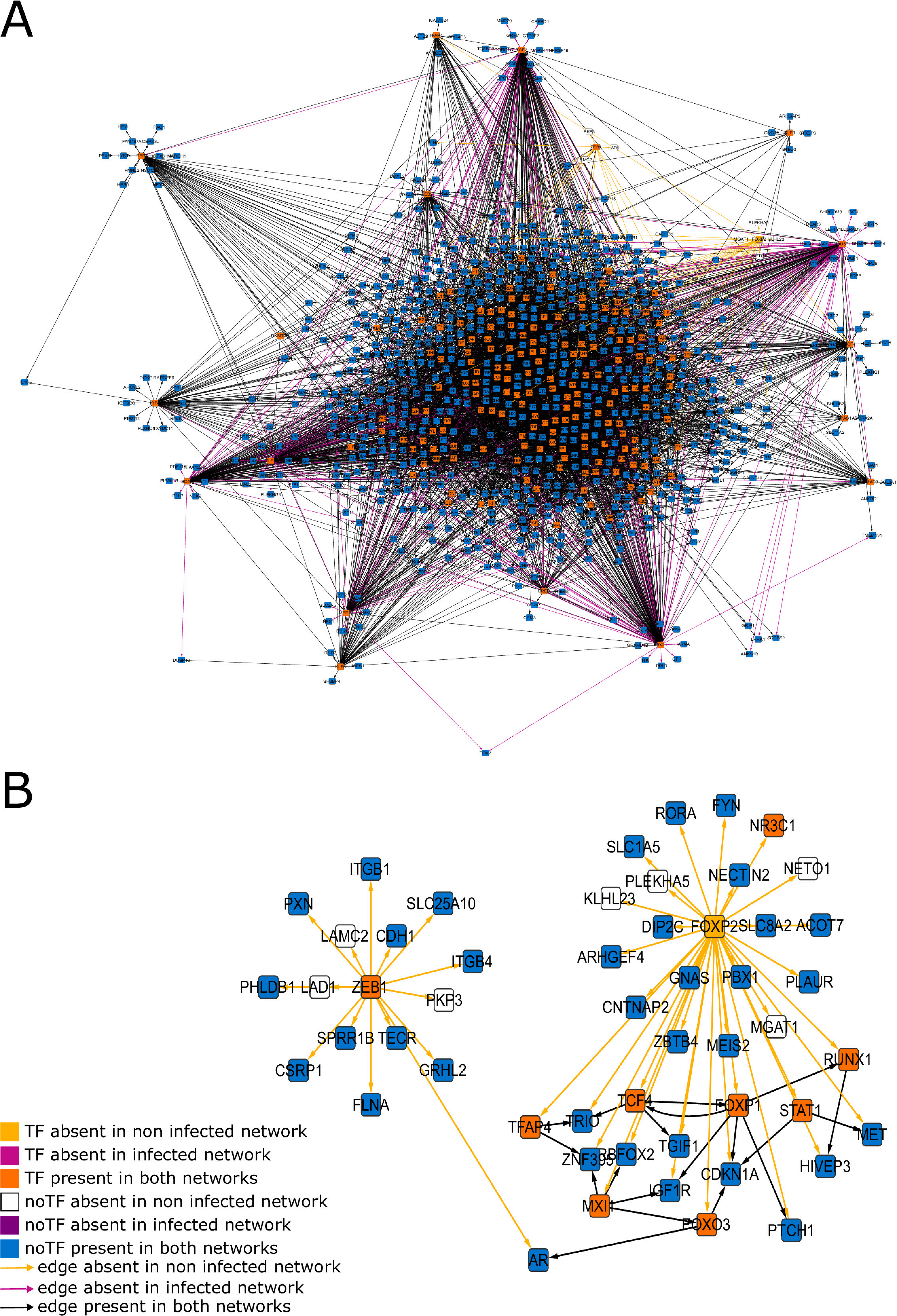
A
B
ITGB1
PXN
SLC25A10
LAMC2
CDH1
ITGB4
PHLDB1
LAD1
ZEB1
PKP3
SPRR1B
TECR
CSRP1
GRHL2
FLNA
FYN
RORA
NR3C1
SLC1A5
NECTIN2
NETO1
PLEKHA5
KLHL23
DIP2C
FOXP2
SLC8A2
ACOT7
ARHGEF4
GNAS
PBX1
PLAUR
CNTNAP2
MGAT1
ZBTB4
MEIS2
RUNX1
TCF4
FOXP1
STAT1
TFAP4
TRIO
MET
TGIF1
ZNF395
RBFOX2
HIVEP3
CDKN1A
IGF1R
MXI1
FOXO3
PTCH1
AR
TF absent in non infected network
TF absent in infected network
TF present in both networks
noTF absent in non infected network
noTF absent in infected network
noTF present in both networks
edge absent in non infected network
edge absent in infected network
edge present in both networks
